# A multi-component intervention increased access to smoking cessation treatment after hospitalization for atherosclerotic cardiovascular disease: a randomized trial

**DOI:** 10.1093/ehjopen/oeae028

**Published:** 2024-04-08

**Authors:** Karin Pleym, Toril Dammen, Harald Wedon-Fekjær, Einar Husebye, Elise Sverre, Serena Tonstad, John Munkhaugen

**Affiliations:** Department of Medicine, Drammen Hospital, Vestre Viken Trust, Dronninggata 48, 3004 Drammen, Norway; Department of Behavioural Medicine, Faculty of Medicine, University of Oslo, Sognsvannsveien 9, 0372 Oslo, Norway; Division of Mental Health and Addiction, Department of Research and Innovation, Oslo University Hospital, Sognsvannsveien 2, 0372 Oslo, Norway; Institute of Clinical Medicine, Faculty of Medicine, University of Oslo, Klaus Torgårds vei 3, 0372 Oslo, Norway; Oslo Center for Biostatistics and Epidemiology, Research Support Services, Oslo University Hospital, Sognsvannsveien 9, 0372 Oslo, Norway; Department of Medicine, Drammen Hospital, Vestre Viken Trust, Dronninggata 48, 3004 Drammen, Norway; Department of Medicine, Drammen Hospital, Vestre Viken Trust, Dronninggata 48, 3004 Drammen, Norway; Department of Endocrinology, Obesity and Preventive Medicine, Section of Preventive Cardiology, Oslo University Hospital, Trondheimsveien 235, 0586 Oslo, Norway; Department of Medicine, Drammen Hospital, Vestre Viken Trust, Dronninggata 48, 3004 Drammen, Norway; Department of Behavioural Medicine, Faculty of Medicine, University of Oslo, Sognsvannsveien 9, 0372 Oslo, Norway

**Keywords:** Smoking, Smoking cessation drugs, Atherosclerotic cardiovascular disease, Motivational interviewing, Secondary prevention, Pro-active referral

## Abstract

**Aims:**

To evaluate the effects of a multi-component intervention for smokers hospitalized for atherosclerotic cardiovascular disease (ASCVD) on the participation rate in community-based cessation programmes and the use of cessation drugs. Additionally, to explore the impact on the cessation rates at 6 months.

**Methods and results:**

A randomized parallel-group study was conducted at a Norwegian secondary care hospital in 2021. The intervention group was: (i) counselled using motivational interviewing techniques during hospitalization; (ii) given an information leaflet, detailing the cessation programme; and (iii) referred to the community-based smoking cessation treatment including a post-discharge pro-active telephone invitation. The control group received usual care and the same information leaflet containing clear contact details for initiating participation. Data were collected at baseline, 1, 3, and 6 months. Among 99 smokers hospitalized with ASCVD, 40 were excluded. Of 59 randomized patients, 4 were lost to follow-up and 55 completed the study. The mean age was 65.1 (standard deviation 9.3) years, 35% were female, and 88% had smoked >20 years. Co-morbidity was prevalent (mean Charlson score 4.8). The intervention group was more likely to participate in the smoking cessation treatment {48 vs. 7%, difference: 41% [95% confidence interval (CI): 14%, 63%]} and used cessation drugs more frequently [59 vs. 21%, difference: 38% (95% CI: 17%, 59%)]. At the 6 months point prevalence, we observed notable between-group differences in self-reported cessation rate (48 vs. 25%).

**Conclusion:**

The intervention significantly increased the participation rate at community-based smoking cessation programmes and the use of cessation drugs among multi-morbid smokers hospitalized for ASCVD.

## Graphical Abstract

**Graphical Abstract:**
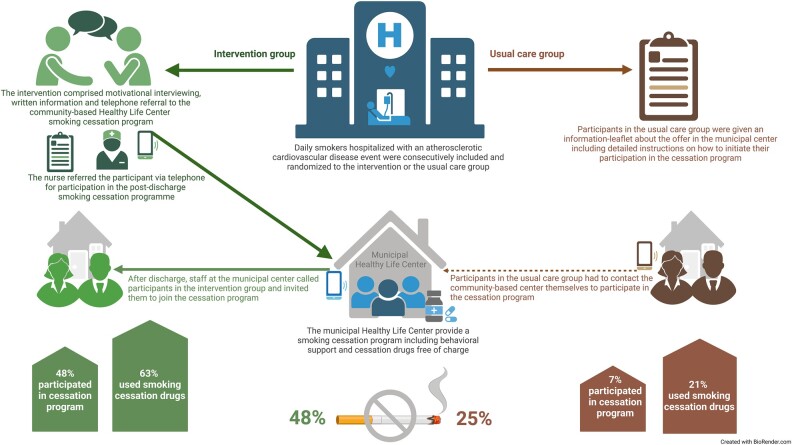

## Introduction

Cigarette smoking is a well-established preventable risk factor for a multitude of diseases, including atherosclerotic cardiovascular disease (ASCVD).^1^ The costs related to smoking are considerable for individuals, the healthcare system, and society.^2^ While the prevalence of daily smokers has declined to ∼8% in the general population in Norway,^3^ smoking continues to be prevalent among patients with ASCVD. In a study from clinical practice, we found that 35% of patients with an acute ASCVD event were daily smokers at the time of the event.^4^ Furthermore, almost 60% continued to smoke, and only 40% reported having received any form of cessation aids, corresponding to recent European surveys.^5^

Hospital-based multi-disciplinary cardiac rehabilitation is recommended following an ASCVD event.^6^ However, due to low accessibility, only 14% of myocardial infarction patients in Norway participate in cardiac rehabilitation.^7,8^ This calls for the development of alternative post-hospitalization strategies. Norwegian health authorities established community-based Healthy Life Centers (HLC) as part of the primary healthcare services across the country. The aim of these centres is to facilitate healthy lifestyle changes.^9^ Patients can either initiate contact themselves or obtain a referral from healthcare professionals for participation. In 2019, ∼30 000 individuals enrolled in various HLC courses.^9^ Nonetheless, only 800 individuals participated in the HLC smoking cessation programmes,^9^ suggesting a gap in reaching the target audience.

Hospitalization following an ASCVD event may act as a motivator for cessation, thus presenting healthcare providers with a valuable opportunity to offer smoking cessation counselling.^10–12^ However, as highlighted by the European Preventive Guidelines, we need a better understanding of how to incorporate successful smoking cessation interventions into routine clinical practices.^1^ A combination of behavioural support and pharmacotherapy is recognized as an effective treatment combination to promote smoking cessation.^13^ Nevertheless, many smokers express scepticism towards the efficacy of cessation drugs and often attempt to quit on their own terms.^14–16^ Additionally, low socioeconomic status, which is prevalent among smokers, may further exacerbate barriers to cessation treatment.^17–19^ In this context, motivational interviewing techniques, with its non-judgemental and empathetic approach, may serve as a beneficial communication method to overcome these challenges.^20,21^

Given the complexities of smoking cessation, we designed a novel, multi-component intervention that combines motivational counselling with information provision during the patient’s hospital stay, coupled with a pro-active referral approach to community-based smoking cessation programmes. The primary objective of this study was to evaluate the effect of this novel intervention on the participation rate at community-based smoking cessation treatment and the utilization of cessation drugs within 6 months after hospitalization. Additionally, we aimed to explore its impact on the smoking cessation rate at the 6-month point prevalence.

## Methods

### Design

This was a prospective, single-centre, randomized, open-label, parallel-group blinded end-point evaluation intervention study (PROBE). Eligible patients were randomly allocated in a 1:1 ratio to either the intervention or the control group utilizing non-transparent envelopes for group assignment. The envelopes were made by an independent statistician and a healthcare provider outside of the research group. The trial was registered at ClinicalTrials.gov (Identifier: NCT04772144) before patient inclusion. No significant changes of methods or outcomes were added after study start. The study was evaluated by the Regional Committee for Medical Research Ethics (202686) and approved by the local data protection officer at Vestre Viken Hospital Trust (PVO-21/07103-1/005). All patients gave written informed consent.

### Participants

A trained cardiac nurse consecutively recruited patients from a Norwegian secondary care hospital from January to November 2021. The study nurse screened patients on all weekdays during this period to assess their eligibility. Patients over 18 years old, who smoked at least one cigarette daily upon hospital admission for an acute ASCVD event (coronary heart disease, peripheral artery disease, or established atherosclerosis and unplanned hospitalization for heart failure, angina, or arrhythmias) and were able and willing to sign informed consent, were included. The inclusion was regardless of their motivation or readiness to quit at baseline. We excluded patients with conditions that could significantly endanger participants, such as psychosis, alcohol abuse, or dementia, or make participation unethical based on the study staff’s assessment. Additionally, individuals with a life expectancy of <12 months due to severe illness, those with an allergy to varenicline, and patients unable to read and understand Norwegian were also excluded.

### Organization of post-discharge care in Norway

The primary care physician holds the main responsibility for secondary preventive care, including cessation counselling, after hospital discharge in Norway.^22^ All ASCVD patients discharged from Drammen Hospital are encouraged to consult their primary physician. Selected patients, particularly those with coronary heart disease, may be referred to cardiac rehabilitation programmes. Primary care community-based HLCs have been available in the hospital’s catchment area since 2006. Smoking cessation follow-up in these community-based centres is flexible and can be conducted in various forms: in-person one-on-one, via video link, telephone, or in groups, according to the patient’s preference. Smoking partners and other family members may also attend the cessation programme if they wish to quit. The cessation programme follows a comprehensive manual developed by the Directorate of Health,^23^ but each centre can adjust the programme to their needs and capacity. Importantly, the offer within the HLC cessation programme is not limited to the study groups, but also available to the general public committing to quit smoking. A standard group-course consists of five to seven sessions and all participants receive a ‘smoking diary’ also containing written information about the recommended use of cessation drugs. Follow-up calls may be conducted if requested. Since 2020, participants have been offered treatment with cessation drugs free of charge. The community-based centres provide patients with a ‘value coupon’, redeemable at pharmacies, covering the cost of either varenicline or a combination of short-acting and long-acting over-the-counter nicotine replacement therapies for a 3-month period. Each participant may receive three ‘value-coupons’. The staff at the community-based Healthy Life Centres have been trained in motivational interview techniques and have learned how to employ the structured cessation programme including online lectures on the appropriate use of cessation drugs.

### Intervention

#### Intervention group

The intervention included nurse-led smoking cessation counselling utilizing motivational interviewing techniques during the patients’ hospital stay. Motivational interviewing is a counselling approach designed to aid individuals in overcoming ambivalent feelings towards behavioural change.^20^ This person-centred methodology focuses on empathy, active listening, and encouragement over confrontation or persuasion, making it particularly suitable for cessation interventions.^21^ A study nurse, not affiliated with the patients’ regular care team, conducted the interviews in single sessions lasting ∼30 min. Prior to taking on this role, the study nurse completed an 18 h training course in motivational interviewing techniques. Moreover, the study nurse received educational feedback on five transcribed patient conversations from the Norwegian expert centre KOURS.^24^ The same study nurse conducted all the motivational interviewing counselling. Cessation counselling was provided to every patient in the intervention group, regardless of their self-reported motivation to quit smoking at baseline. Alongside the cessation counselling, the participants received a standardized information leaflet detailing the community-based smoking cessation offer. Importantly, the intervention included an opt-out telephone referral system, streamlining connections to the community-based smoking cessation treatment. After the patients’ hospital discharge, HLC staff proactively initiated telephone contact with the participants within a 2-week timeframe, actively encouraging their participation in the cessation programme at the community-based HLC. Participation in the HLC programme was optional, and only those attending received cessation drugs free of charge.

#### Control group

The control group received the usual care and the same information leaflet that was developed by the Directorate of Health in Norway, which describes the community-based cessation programme offer. Enrolment instructions with contact information, including telephone and email were clearly visible on the information leaflet. Participants were required to initiate contact with the community-based centre themselves to participate in the cessation programme. Similar to the intervention group, only those who decided to participate in the HLC cessation programme received cessation drugs free of charge.

#### Data collection

At the time of inclusion, we collected data from hospital medical records and self-report questionnaires. The baseline questionnaire was distributed to the participants during hospital stay and was most commonly completed on the day of discharge or the day before. Hospital record data included information about the qualifying ASCVD event, age, sex, co-morbidity, and risk factors. The questionnaires included education, marital status, physical activity (frequency, intensity, and duration), alcohol consumption (≥/<2–3 times/week), the number of years of smoking, previous quit attempts, and smoking partner.

Nicotine addiction was measured with the Fagerström Test for Nicotine Dependence, a six-item questionnaire that quantifies the nicotine dependency by examining time to first cigarette in the morning, abstinence difficulty, preferred cigarette, daily usage, increased morning use, and illness smoking habits. Scores range from 0 to 10, with higher values indicating stronger dependency.^25,26^

Motivation to quit was reported on a 0 (= not motivated) to 10 (= very motivated) Likert scale. Readiness for cessation was assessed by the modified stage of change algorithm;^27^ pre-contemplation (no plans to quit in the next 6 months), contemplation (considering quitting within the next 6 months), or preparation (planning to quit within the next 30 days and having had a successful quit attempt of at least 24 h in the past year).^28^

Study staff blinded to the treatment allocation performed structured telephone interviews at 1, 3, and 6 months after randomization to assess participation at municipal centre (yes vs. no), use of cessation drugs (varenicline, short- or long-acting nicotine replacement therapy), and smoking status, with a current smoker being defined as someone who smoked more than five cigarettes since the start of the cigarette abstinence period.^29^ Self-reported participation and the number and types of consultations were verified through medical records at the community-based HLCs. In line with the Russel Standard,^29^ individuals failing to respond to our telephone call attempts, made on three separate occasions in different days, at the point prevalence of 1, 3, and 6 months, were categorized as smokers.

#### Outcomes

Our two primary endpoints were differences between the control and the intervention groups regarding: (i) participation rate at the cessation programme, obtained from medical records at the municipal centres within 6 months after enrolment in the study and (ii) patient self-report regarding the use of cessation drugs within 6 months after enrolment in the study. The exploratory outcome was the difference in the self-reported prevalence of the smoking cessation rate at the 6-month follow-up.

#### Choice of sample size

We considered a between-group difference in cessation rate at 6 months of 20% to be realistic. Based on previous studies of cessation programmes,^30,31^ ∼50% of programme participants successfully quit. Therefore, we required a difference in participation rate at the HLC programme of at least 40%. A comparable recent study^32^ reported a participation rate of 68% following pro-active telephone contact. As all patients in our study had experienced an acute ASCVD event, we anticipated a 20% participation also in the control group. With 46 (23 × 2) participants, we have 90% power for detecting a between-group difference of 45% (65 vs. 20%) at a 5% significance level using a χ^2^ test. To account for dropouts and an even higher participation rate at the HLC programme in the control group, we decided to randomize 56 patients.

#### Statistical analyses

Continuous variables were described using mean and standard deviation (SD), while categorical variables were described by numbers and percentages. When comparing percentages, confidence intervals (CIs) were calculated by the Agresti–Caffo adjustment (normal distribution with one event and two cases added in each group). The primary analysis included all randomized patients except three who died during the first 2 months before they had a fair chance to enter the community-based cessation programme. A *P*-value of <0.05 was considered statistically significant in all analyses. In the sensitivity analysis, the three patients who died were counted as non-participants in the cessation programme and not using cessation drugs. Data were analysed using SPSS Statistics version 28 (IBM Corp., Armonk, NY, USA) and R (R Foundation for Statistical Computing, Austria).

## Results

**Figure 1:**
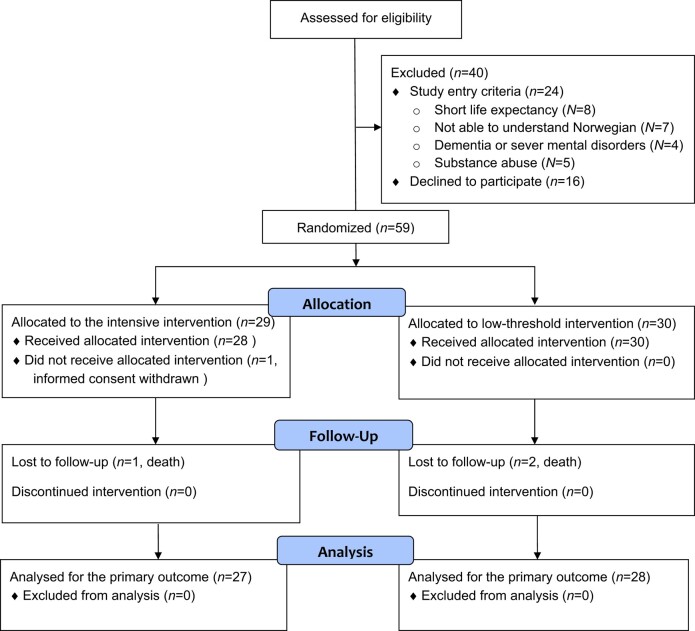
CONSORT flow diagram.^33^

Assessing patients for possible eligibility, 99 patients were identified (*Figure 1*). Of these, 40 were excluded of whom 16 declined to participate. One participant withdrew consent after randomization, leaving 58 patients for randomization to the intervention or control group. Three participants died during follow-up. Accordingly, primary outcome data were available for 55 participants.

The mean age was 65 years, and 35% were female (*Table 1*). Almost 90% had low education indicated by only completion of primary and secondary school. The mean Charlson Comorbidity score was 4.8. The majority (88%) had been smoking for more than 20 years. Motivation for quitting was high with mean 8.5 on a 0 (low) to 10 (high) Likert scale.

**Table 1:** Baseline characteristics of all randomized participants according to treatment allocation

	Intervention group (*n* = 28)	Control group (*n* = 30)	Total (*n* = 58)
Age, mean (SD)	62.8 (9.5)	67.1 (8.7)	65.1 (9.3)
Female, *n* (%)	10 (36)	10 (33)	20 (35)
Low education^a^, *n* (%)	25 (89)	25 (86)	50 (88)
Living alone, *n* (%)	11 (39)	10 (35)	21 (37)
Index cardiovascular event			
Myocardial infarction, *n* (%)	16 (57)	11 (37)	27 (47)
Other atherosclerotic cardiovascular diseases, *n* (%)	12 (43)	19 (63)	31 (53)
Somatic co-morbidity and risk factors			
Chronic obstructive pulmonary disease, *n* (%)	5 (18)	10 (33)	15 (26)
Chronic kidney disease, *n* (%)	3 (11)	4 (13)	7 (12)
Diabetes mellitus, *n* (%)	6 (21)	7 (23)	13 (22)
Hypertension, *n* (%)	19 (68)	23 (77)	42 (74)
Physical inactivity^b^, *n* (%)	15 (54)	18 (62)	33 (58)
Alcohol consumption ≥2–3 times/week, *n* (%)	5 (18)	7 (24)	12 (21)
Charlson co-morbidity sum score, mean (SD)	4.6 (1.8)	5.2 (2.0)	4.8 (1.9)
Smoking frequency, history, and motivation			
Duration of smoking >20 years, *n* (%)	24 (87)	26 (90)	50 (88)
Smoking 1–10 cigarettes per day, *n* (%)	14 (50)	10 (33)	24 (41)
Smoking 11–20 cigarettes per day, *n* (%)	11 (39)	13 (43)	24 (41)
Smoking >20 cigarettes per day, *n* (%)	4 (14)	3 (10)	7 (12)
Living with a smoking partner, *n* (%)	8 (29)	8 (28)	16 (28)
Previous quit attempt, *n* (%)	17 (61)	24 (83)	41 (72)
Motivation to quit^c^, mean (SD)	8.4 (2.1)	8.6 (1.7)	8.5 (1.9)
Readiness for smoking cessation^d^			
Preparation phase, *n* (%)	13 (46)	11 (38)	24 (42)
Contemplation phase, *n* (%)	9 (32)	13 (45)	22 (39)
Pre-contemplation phase, *n* (%)	6 (21)	5 (17)	11 (19)
Nicotine dependency^e^			
Fagerstroms test, mean score (SD)	3.9 (2.0)	4.0 (2.3)	3.9 (2.1)

SD, standard deviation.

^a^Low education was defined by completion of primary and secondary school only.

^b^Physical activity ≤1 time a week.

^c^Motivation to quit was assessed through the baseline questionnaire using a Likert scale ranging from 0 (low motivation) to 10 (high motivation).

^d^Readiness to quit smoking was measured using a modified stage of change algorithm, categorizing individuals into pre-contemplation (no intent to quit within 6 months), contemplation (thinking about quitting in the next 6 months), or preparation (intends to quit in the next 30 days with a past quit attempt of at least 24 h)^29^.

^e^Nicotine dependency assessed by Fagerströms test,^25^ a six-item questionnaire with scores ranging from 0 to10 were low = 0–3, moderate = 4–6, and high = 7–10.

**Figure 2:**
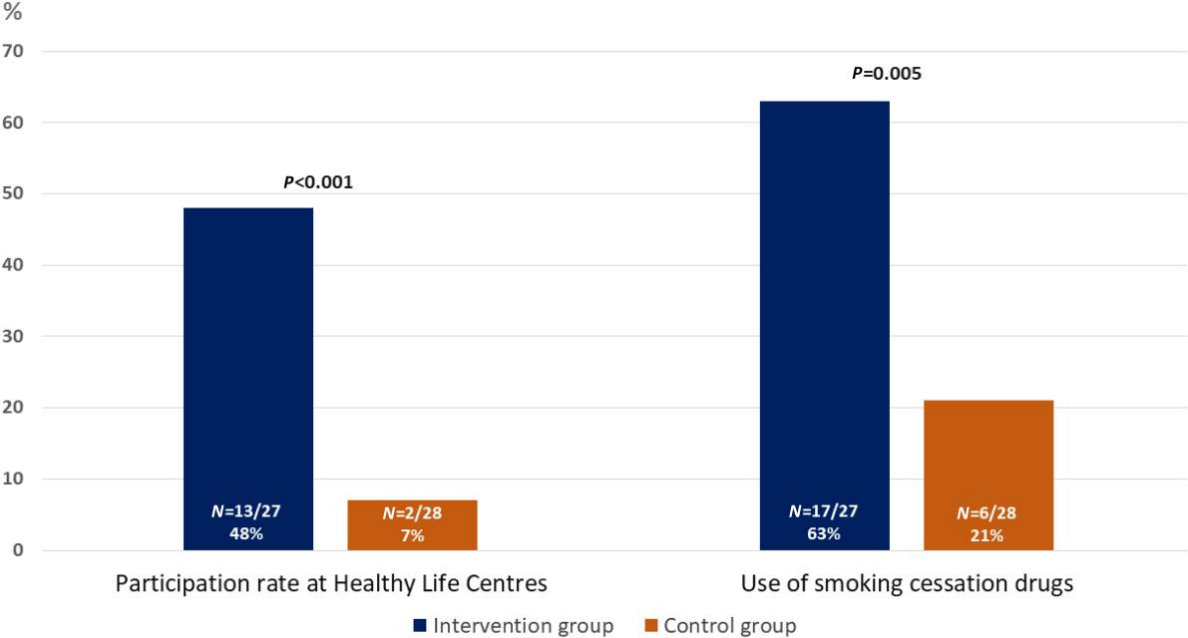
Between-group differences in participation rates at community-based cessation programmes and use of cessation drugs.

The intervention resulted in significantly higher participation rate in the cessation programme compared with the control group with 48 vs. 7%, equalling a 41% percentage points difference (95% CI: 14%, 63%) (*Figure 2* and *Table 2*). Among those participating in the municipal cessation programme, 40% (6/15) engaged in a digital group-course conducted via video call. Another 40% (6/15) had one initial individual consultation in-person followed by subsequent individual telephone calls, whereas 13% (2/15) received only telephone calls. The remaining 7% (1/15) had solely in-person consultations at the municipal centre. The mean total number of digital or physical consultations was 4.4 (SD 1.1).

**Table 2:** Primary and exploratory outcomes^a^

	Intervention group (*n* = 27)	Control group (*n* = 28)	Total (*n* = 58)
*Primary outcomes*			
Participated in a municipal smoking cessation programme, *n* (%)	13 (48.1)	2 (7.1)	15 (27.3)
Use of smoking cessation drugs *n* (%)	16 (59.3)	6 (21.4)	22 (40.0)
Varenicline, *n* (%)	4 (14.8)	3 (10.7)	7 (12.7)
Short- and/or long-acting NRT, *n* (%)	12 (44.5)	3 (10.7)	15 (27.2)
*Exploratory outcomes*			
Self-reported smoke-free at 1 month, *n* (%)	17 (62.9)	11 (39.3)	28 (50.9)
Self-reported smoke-free at 3 months, *n* (%)	12 (44.4)	9 (32.1)	21 (38.2)
Self-reported smoke-free at 6 months, *n* (%)	13 (48.1)	7 (25.0)	20 (36.4)

NRT, nicotine replacement therapy.

^a^The primary outcomes analyses included 55 participants instead of the initial 58, as three participants passed away shortly after randomization and were therefore excluded from these analyses.

Cessation drugs were more frequently used in the intervention group vs. the control group [59 vs. 21%, difference: 42% (95% CI: 8%, 62%)] (*Figure 2* and *Table 2*). Self-reported cessation rate at 6-month follow-up was 48% in the intervention group vs. 25% in the control group equalling a 23% points difference (95% CI: −6%, 49%) (*Table 2*).

Additional sensitivity analyses, counting the three participants lost to follow-up due to death before 2 months after randomization (one in the intervention group and two in the control group), as ‘not participated in the cessation programme’ and as ‘not using cessation drugs’, did not change the primary or exploratory outcomes significantly (data not shown).

In a *post hoc* analysis, we compared the cessation rate at 6 months follow-up in the total population among those who attended at least one visit at the cessation programme to those who did not attend (see Supplementary material online, *Table S1*). Sixty per cent (9/15) self-reported to have quit smoking among the participants compared with 28% (11/40) of the non-participants. Those who participated were more often prepared for cessation (63 vs. 33%) and used more cessation drugs (93 vs. 23%) compared with those who did not attend (*n* = 39).

## Discussion

A multi-component nurse-led intervention for smokers hospitalized with an ASCVD event effectively increased participation in community-based cessation programmes and the use of cessation drugs. Notably, the rate of cessation at 6-month follow-up was nearly doubled, underscoring the potential in implementation of this intervention. Quitting smoking is a challenging process. Thus, breaking down the cessation process into focused interventions for specific tasks can be helpful. Our findings highlight the effectiveness of interventions that target potential barriers smokers may face in seeking, finding, and engaging with relevant health resources for smoking cessation support. Furthermore, the study reveals the positive potential of co-ordinated efforts between hospital and primary care for facilitating access to evidence-based tobacco dependency treatment.

To the best of our knowledge, no previous studies have tested the effect of an intervention for patients hospitalized with an ASCVD event comprising motivational interviewing during hospital stay, telephone referral, and pro-active telephone invitation to community-based cessation programmes with access to free cessation drugs. The intervention was conducted in a routine clinical setting regardless of age, co-morbidity, and underlying ASCVD diagnosis. In a Cochrane review from 2012, the authors concluded that increased personal contact and pro-active referral approaches are more effective in recruiting patients to cessation programmes compared with passive information.^34^ Even though few of the included studies were conducted in a hospital setting, these results correspond well with our findings. Subsequent studies conducted within primary healthcare settings has shown that personalized measures, such as a tailored letter from the general practitioners, are superior to generalized campaigns in recruiting individuals to tobacco dependency treatment.^35^ Our findings align with studies testing the ‘Ask, Advice, Connect’ approach, where patients are referred to a cessation programme by their general practitioner and subsequently contacted by programme staff for participation.^36^ In the ‘Ask, Advice, Connect’ study, they found an increased enrolment in the cessation programme of 14.7 vs. 0.5%.^36^ Our study, adding a motivational interviewing to advice during the hospital stay, demonstrated an even stronger effect on participation, amounting to 48% compared with 7% for the controls receiving passive written information. Additionally, our intervention resulted in significantly more use of cessation drugs, an important element in facilitating successful long-term cessation.^13,37^ The risk of relapse after smoking cessation is indefinite. However, a recent study found that nearly 80% of relapses occurred before 6 months.^38^ A study from Sweden, a context similar to Norway with low smoking rates in the general population and strict tobacco regulations, indeed demonstrated increased long-term cessation success from 1 year to the 5–8 year follow-up.^39^

Ensuring the availability of cessation drugs regardless of patients’ economic status and providing information on correct usage appears to be important elements of our intervention.

Most smokers in our study had low socioeconomic status, which in turn is associated with low health literacy.^4,19^ A recent survey documented that one-third of Norwegians do not understand health information, while half struggle with navigating among various healthcare offers.^40^ The effect of our intervention, related to the pro-active referral approach, the motivating interview, or both, may be mediated by addressing these barriers. Importantly, education levels were comparable between participants and non-participants at the community-based cessation programme, giving no reason to assume that differences in education level influenced participation rate in our study group. Along these lines, a recent systematic review found that pro-active referral resulted in higher enrolment rate at behavioural cessation programmes in a variety of healthcare setting, especially among smokers with low socioeconomic status.^41^

Participants in both the intervention and the control groups self-reported high motivation, and 42% were in the preparation phase, meaning that they already had decided to change behaviour when they responded to the baseline study questionnaire. As expected, more patients were prepared for cessation in the group who attended the community-based cessation programme compared with those who did not. On the other hand, a significant number of patients in the pre-contemplation and contemplation phases also attended the cessation programme. In a qualitative sub-study of our population, we recently found that the non-judgemental patient-centred approach of motivational interviewing was reported as crucial by patients participating in the intervention.^16^ Many participants were elderly and had multiple comorbidities, which may explain why not even more patients attended the community-based smoking cessation programme.

A study from 2022 revealed that the uptake of pharmacotherapies for cessation was poor following hospitalization for an ASCVD event.^42^ In our study, we found large differences between the intervention and the control groups concerning the usage of cessation drugs (59 vs. 21%). Almost all participants who used cessation drugs also attended the community-based cessation programme that provided cessation drugs free of charge. Accordingly, a study from Denmark found that participants receiving subsidized smoking cessation drugs were more likely to complete a cessation course and successfully quit smoking, compared with other participants.^43^

Our results underscore that high motivation, an offer of access to free cessation drugs, and provision of an information leaflet with clearly visible contact information are not sufficient measures to promote participation in smoking cessation programmes following hospitalization for an ASCVD event. These findings correspond with previous literature^41^, and suggest that permanent implementation of a pro-active recruitment strategy to post-discharge cessation programmes for this population would be beneficial. As the intervention builds on existing structures in the Norwegian healthcare system, it will be easy to implement in clinical practice. Our findings are likely relevant to patients hospitalized with various diagnoses, encompassing different lifestyle factors. For firm conclusions, the observed promising effects on cessation rate require a larger study. Therefore, a multi-centre study comprising a health-economic evaluation of the intervention has been initiated and is currently ongoing (NCT05049174).

### Limitations

This was a single-centre study with one nurse conducting all of the motivational interviewing sessions. Therefore, the particular personal skills of the nurse influence the generalization of the results. Studies testing different individuals and healthcare professionals are needed to delineate the critical competence for the motivational interviewing. There was a small, although unexpected age difference between the intervention and control groups, which is most likely due to chance, as we strictly adhered to the randomization protocol. The Hawthorne effect is always a possible source of bias in unblinded studies. However, this risk is mitigated since the control group also received additional care compared with standard clinical practice by receiving written information about the offer as well as the follow-up calls for data collection at 1, 3, and 6 months after discharge. Finally, despite a quite high (60%) participation rate, we excluded non-Norwegian-speaking patients, which limits the generalizability to these patient subgroups.

## Conclusion

A multi-component intervention for smokers hospitalized with an ASCVD event, significantly increased participation in community-based smoking cessation programmes and increased use of smoking cessation drugs. We observed promising outcomes regarding cessation rate after 6 months which encourages and necessitates further research in a larger study. Our study highlights the effectiveness of intervention strategies to overcome individual and system barriers smokers may experience when needing to access and engage with health resources that provide support for smoking cessation.

## Supplementary Material

oeae028_Supplementary_Data

## Data Availability

Study data are available on request only, due to privacy/ethical restrictions. The corresponding author should be contacted for data requests.

## References

[1] Visseren FLJ , Mach F, Smulders YM, Carballo D, Koskinas KC, Bäck M, Benetos A, Biffi A, Boavida JM, Capodanno D, Cosyns B, Crawford C, Davos CH, Desormais I, Di Angelantonio E, Franco OH, Halvorsen S, Hobbs FDR, Hollander M, Jankowska EA, Michal M, Sacco S, Sattar N, Tokgozoglu L, Tonstad S, Tsioufis KP, van Dis I, van Gelder IC, Wanner C, Williams B; ESC National Cardiac Societies; ESC Scientific Document Group. 2021 ESC Guidelines on cardiovascular disease prevention in clinical practice. Eur Heart J 2021;42:3227–3337. 34458905 10.1093/eurheartj/ehab484

[2] Ekpu VU , Brown AK. The economic impact of smoking and of reducing smoking prevalence: review of evidence. Tob Use Insights 2015;8:1–35. 26242225 10.4137/TUI.S15628 PMC4502793

[3] Helsedirektoratet . Statistikk og historikk om røyking, snus og e-sigaretter. https://www.helsedirektoratet.no/tema/tobakk-royk-og-snus/statistikk-om-royking-bruk-av-snus-og-e-sigaretter (1 April 2022).

[4] Sverre E , Otterstad JE, Gjertsen E, Gullestad L, Husebye E, Dammen T, Moum T, Munkhaugen J. Medical and sociodemographic factors predict persistent smoking after coronary events. BMC Cardiovasc Disord 2017;17:241. 28877684 10.1186/s12872-017-0676-1 PMC5588720

[5] De Bacquer D , Astin F, Kotseva K, Pogosova N, De Smedt D, De Backer G, Rydén L, Wood D, Jennings C. Poor adherence to lifestyle recommendations in patients with coronary heart disease: results from the EUROASPIRE surveys. Eur J Prev Cardiol 2021;29:383–395. 10.1093/eurjpc/zwab115 34293121

[6] Ambrosetti M , Abreu A, Corrà U, Davos CH, Hansen D, Frederix I, Iliou MC, Pedretti RFE, Schmid JP, Vigorito C, Voller H, Wilhelm M, Piepoli MF, Bjarnason-Wehrens B, Berger T, Cohen-Solal A, Cornelissen V, Dendale P, Doehner W, Gaita D, Gevaert AB, Kemps H, Kraenkel N, Laukkanen J, Mendes M, Niebauer J, Simonenko M, Zwisler AO. Secondary prevention through comprehensive cardiovascular rehabilitation: from knowledge to implementation. 2020 update. A position paper from the Secondary Prevention and Rehabilitation Section of the European Association of Preventive Cardiology. Eur J Prev Cardiol 2021;28:460–495. 33611446 10.1177/2047487320913379

[7] Olsen SJS , Schirmer H, Bønaa KH, Hanssen TA. Cardiac rehabilitation after percutaneous coronary intervention: results from a nationwide survey. Eur J Cardiovasc Nurs 2018;17:273–279. 29048205 10.1177/1474515117737766

[8] Norekvål TM , Bale M, Bedane HK, Hole T, Ingul CB, Munkhaugen J. Cardiac rehabilitation participation within 6 months of discharge in 37 136 myocardial infarction survivors: a nationwide registry study. Eur J Prev Cardiol 2023;00:zwad350. 10.1093/eurjpc/zwad350 37943676

[9] Thonstad M , Ekornrud T, Stølan SBC. Frisklivssentraler og tilsvarende helsefremmende tilbud i norske kommuner 2019. Analyse av tilbud for livsstilsendringer og mestring av sykdom. Kongsvinger, Oslo: Statistisk Sentralbyrå 2020, p. 140.

[10] Rigotti NA , Clair C, Munafò MR, Stead LF. Interventions for smoking cessation in hospitalised patients. Cochrane Database Syst Rev 2012;5:CD001837. 22592676 10.1002/14651858.CD001837.pub3 PMC4498489

[11] Snaterse M , Jorstad HT, Minneboo M, Lachman S, Boekholdt SM, ter Riet G, Scholte Op Reimer WJM, Peters RJG. Smoking cessation after nurse-coordinated referral to a comprehensive lifestyle programme in patients with coronary artery disease: a substudy of the RESPONSE-2 trial. Eur J Cardiovasc Nurs 2019;18:113–121. 30122068 10.1177/1474515118795722

[12] Riley H , Ainani N, Turk A, Headley S, Szalai H, Stefan M, Lindenauer PK, Pack QR. Smoking cessation after hospitalization for myocardial infarction or cardiac surgery: assessing patient interest, confidence, and physician prescribing practices. Clin Cardiol 2019;42:1189–1194. 31647127 10.1002/clc.23272 PMC6906990

[13] Stead LF , Koilpillai P, Fanshawe TR, Lancaster T. Combined pharmacotherapy and behavioural interventions for smoking cessation. Cochrane Database Syst Rev 2016;3:CD008286. 27009521 10.1002/14651858.CD008286.pub3 PMC10042551

[14] Roddy E , Antoniak M, Britton J, Molyneux A, Lewis S. Barriers and motivators to gaining access to smoking cessation services amongst deprived smokers – a qualitative study. BMC Health Serv Res 2006;6:147. 17087825 10.1186/1472-6963-6-147 PMC1647276

[15] van Wijk EC , Landais LL, Harting J. Understanding the multitude of barriers that prevent smokers in lower socioeconomic groups from accessing smoking cessation support: a literature review. Prev Med 2019;123:143–151. 30902700 10.1016/j.ypmed.2019.03.029

[16] Getz V , Munkhaugen J, Lie HC, Dammen T. Barriers and facilitators for smoking cessation in chronic smokers with atherosclerotic cardiovascular disease enrolled in a randomized intervention trial: a qualitative study. Front Psychol 2023;14:1060701. 37034951 10.3389/fpsyg.2023.1060701 PMC10074255

[17] Friis K , Lasgaard M, Rowlands G, Osborne RH, Maindal HT. Health literacy mediates the relationship between educational attainment and health behavior: a danish population-based study. J Health Commun 2016;21:54–60. 27668691 10.1080/10810730.2016.1201175

[18] Svendsen MT , Bak CK, Sørensen K, Pelikan J, Riddersholm SJ, Skals RK, Mortensen RN, Maindal HT, Bøggild H, Nielsen G, Torp-Pedersen C. Associations of health literacy with socioeconomic position, health risk behavior, and health status: a large national population-based survey among Danish adults. BMC Public Health 2020;20:565. 32345275 10.1186/s12889-020-08498-8 PMC7187482

[19] Stormacq C , Van den Broucke S, Wosinski J. Does health literacy mediate the relationship between socioeconomic status and health disparities? Integrative review. Health Promot Int 2018;34:e1–e17. 10.1093/heapro/day062 30107564

[20] Rollnick S , Miller WR, Butler C. Motivational Interviewing in Health Care: Helping Patients Change Behavior. 2nd ed. New York: The Guilford Press; 2022.

[21] Lindson N , Thompson TP, Ferrey A, Lambert JD, Aveyard P. Motivational interviewing for smoking cessation. Cochrane Database Syst Rev 2019;7:CD006936. 31425622 10.1002/14651858.CD006936.pub4 PMC6699669

[22] Peersen K , Munkhaugen J, Gullestad L, Liodden T, Moum T, Dammen T, Perk J, Otterstad JE. The role of cardiac rehabilitation in secondary prevention after coronary events. Eur J Prev Cardiol 2017;24:1360–1368. 28664773 10.1177/2047487317719355

[23] Helsedirektoratet . Booklet. Tobakksfri – Mal for snus- og røykesluttkurs for deg som jobber på frisklivssentralen. 2012. https://www.helsedirektoratet.no/brosjyrer/tobakksfri-mal-for-snus-og-roykesluttkurs-for-deg-som-jobber-pa-frisklivssentralen.

[24] KORUS . KORUS (Kompetansesenter for rusfeltet). https://korus.no/ (21 June 2022).

[25] Fagerström KO . Measuring degree of physical dependence to tobacco smoking with reference to individualization of treatment. Addict Behav 1978;3:235–241. 735910 10.1016/0306-4603(78)90024-2

[26] Sharma MK , Suman LN, Srivastava K, Suma N, Vishwakarma A. Psychometric properties of Fagerstrom test of nicotine dependence: a systematic review. Ind Psychiatry J 2021;30:207–216. 35017802 10.4103/ipj.ipj_51_21 PMC8709504

[27] DiClemente CC , Prochaska JO, Fairhurst SK, Velicer WF, Velasquez MM, Rossi JS. The process of smoking cessation: an analysis of precontemplation, contemplation, and preparation stages of change. J Consult Clin Psychol 1991;59:295–304. 2030191 10.1037//0022-006x.59.2.295

[28] Prugger C , Wellmann J, Heidrich J, De Bacquer D, De Backer G, Périer MC, Empana JP, Reiner Ž, Fras Z, Jennings C, Kotseva K, Wood D, Keil U; EUROASPIRE Study Group. Readiness for smoking cessation in coronary heart disease patients across Europe: results from the EUROASPIRE III survey. Eur J Prev Cardiol 2015;22:1212–1219. 25516535 10.1177/2047487314564728

[29] West R , Hajek P, Stead L, Stapleton J. Outcome criteria in smoking cessation trials: proposal for a common standard. Addiction 2005;100:299–303. 15733243 10.1111/j.1360-0443.2004.00995.x

[30] Quist-Paulsen P , Gallefoss F. Randomised controlled trial of smoking cessation intervention after admission for coronary heart disease. BMJ 2003;327:1254–1257. 14644967 10.1136/bmj.327.7426.1254 PMC286243

[31] Leosdottir M , Wärjerstam S, Michelsen HÖ, Schlyter M, Hag E, Wallert J, Larsson M. Improving smoking cessation after myocardial infarction by systematically implementing evidence-based treatment methods. Sci Rep 2022;12:642. 35022490 10.1038/s41598-021-04634-5 PMC8755785

[32] Vidrine JI , Shete S, Cao Y, Greisinger A, Harmonson P, Sharp B, Miles L, Zbikowski SM, Wetter DW. Ask-Advise-Connect: a new approach to smoking treatment delivery in health care settings. JAMA Intern Med 2013;173:458–464. 23440173 10.1001/jamainternmed.2013.3751 PMC3858085

[33] Schulz KF , Altman DG, Moher D. CONSORT 2010 Statement: updated guidelines for reporting parallel group randomised trials. BMC medicine 2010;8:18–NaN. 10.1136/bmj.c332 20334633 PMC2860339

[34] Belisario JSM , Bruggeling MN, Gunn LH, Brusamento S, Car J. Interventions for recruiting smokers into cessation programmes. Cochrane Database Syst Rev 2012;12:CD009187. 23235672 10.1002/14651858.CD009187.pub2 PMC6485998

[35] Gilbert H , Sutton S, Morris R, Petersen I, Wu Q, Parrott S, Galton S, Kale D, Magee MS, Gardner L, Nazareth I. Start2quit: a randomised clinical controlled trial to evaluate the effectiveness and cost-effectiveness of using personal tailored risk information and taster sessions to increase the uptake of the NHS Stop Smoking Services. Health Technol Assess 2017;21:1–206. 10.3310/hta21030 PMC5292643 28121288

[36] Vidrine JI , Shete S, Li Y, Cao Y, Alford MH, Galindo-Talton M, Rabius V, Sharp B, Harmonson P, Zbikowski SM, Miles L, Wetter DW. The Ask-Advise-Connect approach for smokers in a safety net healthcare system: a group-randomized trial. Am J Prev Med 2013;45:737–741. 24237916 10.1016/j.amepre.2013.07.011 PMC4023543

[37] Hartmann-Boyce J , Chepkin SC, Ye W, Bullen C, Lancaster T. Nicotine replacement therapy versus control for smoking cessation. Cochrane Database Syst Rev 2018;5:CD000146. 29852054 10.1002/14651858.CD000146.pub5 PMC6353172

[38] Acar T , Gallagher C, Gören Y, Erbas B, Özkara A. Determinants of smoking cessation outcomes and reasons for relapse in patients admitted to a smoking cessation outpatient clinic in Turkey. Int J Environ Res Public Health 2024;21:310. 38541309 10.3390/ijerph21030310 PMC10970556

[39] Nohlert E , Öhrvik J, Tegelberg Å, Tillgren P, Helgason ÁR. Long-term follow-up of a high- and a low-intensity smoking cessation intervention in a dental setting–a randomized trial. BMC Public Health 2013;13:592. 23777201 10.1186/1471-2458-13-592 PMC3693879

[40] Le C , Finbråten HS, Pettersen KS, Joranger P, Guttersrud Ø. Befolkningens helsekompetanse, del I. The International Health Literacy Population Survey 2019–2021 (HLS19) – et samarbeidsprosjekt med nettverket M-POHL tilknyttet WHO-EHII. Rapport IS-2959. 2021.

[41] van Westen-Lagerweij NA , Hipple Walters BJ, Potyka F, Croes EA, Willemsen MC. Proactive referral to behavioral smoking cessation programs by healthcare staff: a systematic review. Nicotine Tob Res 2023;25:849–858. 36394282 10.1093/ntr/ntac262 PMC10077936

[42] Robijn AL , Woodward M, Pearson SA, Hsu B, Chow CK, Filion KB, Jorm L, Havard A. Uptake of prescription smoking cessation pharmacotherapies after hospitalization for major cardiovascular disease. Eur J Prev Cardiol 2022;29:2173–2182. 35950363 10.1093/eurjpc/zwac172

[43] Vibjerg H . The Danish model for smoking cessation. Tob Prev Cessation 2021;7:22.

