# Correction to: A multi-component intervention increased access to smoking cessation treatment after hospitalization for atherosclerotic cardiovascular disease: a randomized trial

**DOI:** 10.1093/ehjopen/oeaf010

**Published:** 2025-02-19

This is a correction to: Karin Pleym *et al.*, A multi-component intervention increased access to smoking cessation treatment after hospitalization for atherosclerotic cardiovascular disease: a randomized trial, *European Heart Journal Open*, Volume 4, Issue 2, March 2024, https://doi.org/10.1093/ehjopen/oeae028

In the version originally published, the author Harald Wedon-Fekjær was incorrectly listed as Harald Wedon-Fekjaer. The listing has been corrected.

