# Supplementary material for: A multi-component intervention increased access to smoking cessation treatment after hospitalization for atherosclerotic cardiovascular disease: a randomized trial
Source: Eur Heart J Open. 2024 Apr 8;4(2):oeae028. doi: 10.1093/ehjopen/oeae028 (PMC11044967; doi:10.1093/ehjopen/oeae028)
Supplement: oeae028_Supplementary_Data [file oeae028_supplementary_data.docx]

**Supplementary Table 1**. Clinical characteristics of participants and non-participants at the Healthy Life Centres smoking cessation program

| **Study factors** | **Participants  (n= 16)** | **Non-participants (n=39)** |
| --- | --- | --- |
| Age in years, mean (SD) | 62.1 (8.1) | 65.8 (9.7) |
| Female gender, N (%) | 6 (37.5) | 12 (30.8) |
| Low education, N (%) | 14 (87.5) | 34 (87.2) |
| Charlson score, mean (SD) | 4.3 (1.7) | 4.8 (1.9) |
| Prepared for cessation^a^, N (%) | 10 (62.5) | 13 (33.3) |
| High nicotine dependency, N (%) | 5 (31.0) | 14 (35.8) |
| Randomized to the nurse-led intervention, N (%) | 13 (81.3) | 3 (18.8) |

S.D: Standard deviation

^a^Prepared for smoking cessation assessed by the Transtheoretical Model of Health Behaviour Change, Prochaska (1997).
